# Antarctic Krill *Euphausia superba* Oil Supplementation Attenuates Hypercholesterolemia, Fatty Liver, and Oxidative Stress in Diet-Induced Obese Mice

**DOI:** 10.3390/nu16213614

**Published:** 2024-10-24

**Authors:** Jun-Hui Choi, Se-Eun Park, Seung Kim

**Affiliations:** Department of Food Science and Nutrition, Gwangju University, Gwangju 61743, Republic of Korea; sekai0572@naver.com (J.-H.C.);

**Keywords:** Antarctic krill, *Euphausia superba*, hypercholesterolemia

## Abstract

Background: Several Previous studies indicate that consuming krill oil may aid in reducing hypercholesterolemia and improving cholesterol metabolism. Therefore, our study was designed to investigate the effectiveness of Antarctic krill oil (*Euphausia superba*) (ESKO) in combating obesity and lowering fat/lipid/cholesterol levels. Methods: The study aimed to investigate the molecular docking model targeting 3-hydroxy-3-methylglutaryl-CoA reductase (HMGCR) using ESKO-derived eicosapentaenoic acid (EPA), docosahexaenoic acid (DHA), and astaxanthin. In this study, histological alterations in the liver of the obesity model (ICR male mouse), obesity-related or antioxidant markers in both liver and serum, the molecular mechanisms in HepG2 cells and liver tissue, and HMGCR activity were analyzed. Results: Our findings revealed that a high-fat diet (HFD) significantly led to increased oxidative stress, obesity-related indicators, and cardiovascular-associated risk indices. However, ESKO effectively mitigated HFD-induced oxidative stress, fat accumulation, and the suppression of low-density lipoprotein receptor (LDLR) or activation of related molecular pathways. This was achieved through improvements in metabolic parameters, including CD36/liver X receptor α (LXRα)/sterol regulatory element-binding protein 1c (SREBP1c), proprotein convertase subtilsin/kexin type 9 (PCSK-9), and HMGCR, ultimately ameliorating HFD-induced hypercholesterolemia and obesity. Conclusions: These beneficial findings indicate that ESKO might have significant potential for preventing and treating obesity-related disorders.

## 1. Introduction

Obesity and obesity-associated disorders including hypercholesterolemia and hyperlipidemia are characterized by an energy imbalance resulting from excessive energy intake or insufficient energy expenditure, leading to an accumulation/increase of excessive adipose tissue and several biochemical parameters of serum level in the human body. Although only included in the World Health Organization’s disease code for about 50 years, obesity has swiftly evolved into a global concern, significantly impacting human health conditions and disabilities [1]. If current trends persist, over 4 billion people, representing 51% of the global population, will be affected by either overweight or obesity by 2035, and nearly 2 billion individuals or one in four people will specifically experience obesity [2]. In Korea, the prevalence of obesity has recently surged. The 2020 National Health and Nutrition Survey showed the proportion of individuals aged 19 or older with a body mass index of 25 kg/m^2^ or higher increased by 4.5% to 38.3% in 2020 compared to the preceding year [3], and there is anticipation of a swift increase by the year 2030 [4]. When excess energy accumulates, leading to the disruption of adipose tissue’s normal functions, fat starts accumulating in various tissues such as the liver, muscles, pancreas, and heart, apart from adipose tissue itself. Damaged adipose tissue triggers a shift in macrophages and T cells from an anti-inflammatory to a pro-inflammatory state [5]. The secretion of inflammatory cytokines like interleukin-1β, interleukin-6, and tumor necrosis factor-α, leading to chronic inflammation and insulin resistance [6]. Fat accumulation in the liver promotes the synthesis of neutral fat, which in turn triggers the secretion of very low-density lipoprotein, contributing to insulin resistance in the liver [7]. Furthermore, obesity resulting from fat buildup elevates the risk of developing conditions such as high blood pressure, coronary cardiovascular disease, arthritis, gallbladder disease, and specific cancers [8]. This increased risk of mortality underscores the importance of prevention and management strategies for obesity, which stands as one of the critical diseases to address.

Antarctic krill is a small shrimp-like microcrustacean known as *Euphausia superba* and its primary composition includes moisture (ranging from 77.9% to 83.1%), protein (between 11.9% and 15.4%), lipid (0.4% to 3.6%), ash (3%), carbohydrates (2%), chitin, chitosan (2%), and other glucides [9]. In terms of its lipid content, Antarctic krill stands as one of the richest sources of omega-3 polyunsaturated fatty acids (PUFAs), and it boasts abundant docosahexaenoic acid (DHA) and eicosapentaenoic acid (EPA), which primarily bind to phospholipids (PLs) such as phosphatidylcholine, whereas in fish oil, they predominantly bind to triacylglycerols (TG) [10]. Many studies have indicated that omega-3 PUFA bound to PL are absorbed and integrated into cell membranes more effectively than those bound to TG, suggesting that the omega-3 PUFA found in krill oil offer enhanced bioavailability [11]. Moreover, krill oil boasts significant levels of astaxanthin, a potent antioxidant [12], and has garnered considerable attention as a superior source of omega-3 PUFA with distinct biological effectiveness over recent decades [13,14]. In recent research, several studies have explored the functions of krill oil in various physiological and metabolic responses, as well as its effectiveness in improving cardiovascular disease (CVD) outcomes [15,16,17,18,19]. Therefore, this study extensively assessed the impact of *E. superba* krill oil (ESKO) treatment on high-fat diet-induced obesity mouse models with hypercholesterolemia. The assessment covered various indicators, including lipid accumulation, intracellular triglyceride levels, and 3-hydroxy-3-methylglutaryl-CoA reductase (HMGCR) activity in HepG2 cells, as well as levels of serum lipids and related parameters, liver tissue histology, epididymal fats, in silico molecular docking analysis using ESKO-derived key substances (including EPA, DHA, and astaxanthin) on HMGCR enzyme by simulation software, binding energy scores, and the CD36/LXRα/SREBP1c or LDLR/PCSK9 pathway in liver tissue in the mouse model. This research contributes to enhancing understanding regarding the potential application of ESKO in preventing obesity and hypercholesterolemia-associated symptoms.

## 2. Materials and Methods

### 2.1. Materials

Fenofibrate, trizma base, ethylenediaminetetraacetic acid (EDTA), dimethyl sulfoxide (DMSO), RIPA lysis buffer, and Oil-Red O were purchased from Sigma-Aldrich (St. Louis, MO, USA). Dulbecco’s Modified Eagle Medium (DMEM), fetal bovine serum (FBS), streptomycin, and penicillin were purchased from Invitrogen (Carlsbad, CA, USA). Other reagents were purchased for special grade. Fresh Antarctic *Euphausia superba* krill oil (ESKO) was obtained from RIMFROST NEW ZEALAND LTD (Nelson, New Zealand) on 4 April 2023, QA Manager, Kristin Iversen from RIMFROST NEW ZEALAND confirmed the authenticity of ESKO.

### 2.2. Cell Culture

HepG2 cells sourced from the Korean Cell Line Bank were utilized in the study as previously described [20]. Dulbecco’s Modified Eagle medium (DMEM) added with 1% streptomycin-penicillin and 10% fetal bovine serum (FBS) was employed for cell culture, maintained at 37 °C with 5% CO_2_. Upon reaching approximately 80% confluency, HepG2 cells were subcultured and subjected to serum-free DMEM for 24 h prior to treatment with palmitic acid and ESKO for another 24 h. The Control group received treatment involving the addition of 5% bovine serum albumin to the medium.

### 2.3. Preparation of ESKO and Palmitic Acid Solution

A stock solution of ESKO was prepared at a concentration of 20 mg/mL in ethanol. The preparation of the palmitic acid solution followed a modification of the method outlined by Cousin et al. [21]. Initially, a 100 mM palmitic acid stock solution was created by heating in a 0.1 mM NaOH solution at 70 °C. Subsequently, a 10 mM FFA/1% BSA solution was generated by combining the palmitic acid stock solution with a 10% (*w*/*v*) FFA-free BSA solution held at 55 °C in ddH_2_O, and allowed to react for 30 min. Afterward, the resultant solution was cooled to 25 °C, filtered, stored at −20 °C, and utilized in the experiment.

### 2.4. Cell Viability Assay

HepG2 cells were seeded in a plate with 96-well at a density of 4 × 10^4^ cells per well, then subjected to treatment with palmitic acid (50, 100, 300, 500, 700, 900, and 1000 μM) and ESKO (10, 50, 100, 300, 500, 700, and 1000 μg/mL), and cultured for 24 h as previously described [20]. Following the incubation period, the medium was aspirated, and 10 μL of a 5 mg/mL of 3-(4,5-dimethylthiazol-2-yl)-2,5-diphenyl tetrazolium bromide (MTT) solution was treated, allowing for a 4-h reaction. Upon completion of the reaction, dissolution of the formed formazan crystals was performed with DMSO. The absorbance of the solution was then measured using an ELISA reader at 540 nm.

### 2.5. Oil Red O Staining

HepG2 cells were plated into a 6-well dish, exposed to 0.3 mM palmitic acid and ESKO (50, 100, and 150 μg/mL) for a duration of 24 h, followed by two washes with phosphate buffered saline (PBS), as previously described [20]. Subsequently, the cells were fixed with formaldehyde at room temperature. Post-fixation, a triple wash with PBS was performed, succeeded by staining with Oil Red O staining reagent for 1 h at room temperature. Upon completion of staining, the cells were thoroughly rinsed with distilled water to remove excess Oil Red O staining reagent. Following this, 200 μL of Isopropanol was added to dissolve the intracellular staining reagent, and the absorbance was measured at 520 nm using an ELISA reader.

### 2.6. Triglyceride Level

For the quantification of intracellular triglyceride level, HepG2 cells were seeded at a density of 1 × 10^6^ cells per well in a 6-well plate and exposed to 0.3 mM palmitic acid and ESKO (50, 100, and 150 μg/mL) oil for 24 h, as previously described [20]. Assessment of neutral fat and cholesterol content was conducted utilizing an assay kit following the manufacturer’s provided analysis protocol.

### 2.7. HMG-CoA Reductase Activity Analysis

HepG2 cells were treated with ESKO (50, 100, and 150 μg/mL), and 0.3 mM palmitic acid in a 6-well plate for 16 h, and then HMGCR activity assay kit (Sigma-Aldrich, St. Louis, MO, USA) and was confirmed by performing the analysis according to the analysis method provided by the manufacturer [20].

### 2.8. Molecular Docking

Molecular docking analysis utilized Human HMG-CoA reductase (PDB code: 1HWK), along with DHA (PubChem CID: 445580), EPA (PubChem CID: 446284), astaxanthin (PubChem CID: 5281224), and atorvastatin (PubChem CID: 60823). AutoDock4 [22] and PyMol [23] facilitated the analysis process as previously described [24]. AutoDock Vina 4.2.6 software was employed to predict the binding energy scores between the experimental ligands (astaxanthin, DHA, EPA, and atorvastatin) acting as inhibitors and the protein HMGCR. AutoDock Vina, designed specifically for protein-ligand docking, is a molecular modeling and simulation software [25]. Grid maps covering all residues were formed for molecular binding analysis, with 2,500,000 evaluations and 10 runs. Docking results were computed through the Lamarckian Genetic Algorithm, and AutoDockTools 1.5.6 and PyMol 3.0.0 software were used for visualization of the outcomes. AutoDockTools molecular graphics software facilitated modeling the complex structure of experimental ligands (astaxanthin, DHA, EPA, or atorvastatin) and HMGCR proteins via molecular docking across all amino acid residues within specific distances (Å). Utilizing Discovery Studio Visualizer 2021 software (Accelrys Software Inc., San Diego, CA, USA), images depicting enzyme binding pockets and binding profiles with ligands were generated.

### 2.9. Animals

For the experiment on diet-induced obesity (DIO) mouse models, male Imprinting Control Region (ICR) mice aged 8 weeks and weighing between 30 and 40 g were utilized. These mice were housed in cages accommodating three animals each and were maintained under controlled environmental conditions, with a 12-h light or dark cycle and a temperature range of 22 ± 2 °C. The mice had unrestricted access to either standard feed (LabDiet 5L79; ORIENT BIO Inc., Seongnam, Republic of Korea) or a high-fat diet comprising 60% fat content (TestDiet 58Y1, ORIENT BIO Inc., Seongnam, Republic of Korea), as well as tap water ad libitum. The compositions of the administered diets are presented in Table 1. In order to alleviate animal distress and minimize the usage of animals, all experimental protocols adhered to the guidelines outlined in the National Institutes of Health Guide for the Care and Use of Laboratory Animals (NIH publication no. 80-23, revised 1996) and the relevant ethical regulations of Gwangju University. Furthermore, these experiments were approved on 8 November 2023 by the Institutional Animal Care and Use Committee of Chosun University (CIACUC2023-S0032).

### 2.10. In Vivo DIO Mouse Model and Treatment Groups

The influence of ESKO was examined over a 60-day period on DIO models. These mice were fed a high-fat diet (HFD) continuously for 60 days from day 1 to day 60, and ESKO (400 mg/kg/day, 16 mg/mouse/day) and fenofibrate (200 mg/kg/day, 8 mg/mouse/day) were administered orally simultaneously from day 1 to day 60, respectively. Twenty-four male mice were divided into four groups with six mice per cage, and the sample size is equal to the number of animals, without exception. Group 1 included normal mice fed a standard diet and treated with saline as a vehicle (Control group). Group 2 comprised the DIO model fed the HFD and treated with saline (vehicle) (DIO group). Groups 3 and 4 contain the DIO model administered at doses of 400 mg/kg ESKO (DIO + ESKO group), and 200 mg/kg fenofibrate (DIO + Fenofibrate group), respectively. Throughout the experiment, food intake and body weight (BW) were monitored, and BW reduction (%) was calculated using the formula: 100 − [(final BW − initial BW)/initial BW] × 100. The feed efficiency ratio was determined by dividing the body weight gain (final BW − initial BW) by the mass of feed intake. Upon completion of the study, following a 12-h fasting time, animals in all groups were anesthetized using light ether. Serum was extracted from whole blood collected (2 mL), which was subsequently centrifuged at 1500× *g* for 15 min. The serum samples were then stored at −70 °C for further experimentation. Cervical dislocation was then performed to sacrifice the subjects, allowing for the collection of various tissues, including fat, liver, kidney, and spleen samples for tissue analysis. Protein concentrations were measured utilizing the bicinchoninic acid (BCA) method and bovine serum albumin serving as the standard.

### 2.11. Tissue Analysis and Liver Histology

Following an overnight fasting period, tissues including liver, kidney, spleen, and epididymal fats were collected, and weighed as previously described [4]. Additionally, the liver underwent perfusion with 10% formalin and was fixed for 24 h in 10% formalin prior to analysis. Histological assessment of fat accumulation from the tissue was conducted for color intensity, expressed as a percentage utilizing Oil Red O staining and ImageJ 1.54 [4]. Liver samples were examined using a VENTANA DP600 slide scanner (Roche Diagnostics, Basel, Switzerland). Frozen tissues sectioned through a cryostat were fixed and stained using Oil Red O.

### 2.12. Catalase Assay

Catalase (CAT) activity was evaluated following a modified version of the described method [4]. A solution comprising 1 μM H_2_O_2_, 50 mM sodium phosphate buffer (pH 7.4), and tissue homogenate or serum was prepared and the reduction in absorbance was monitored at 240 nm over a period of 10 m. CAT activity of 1 U corresponded to the enzyme quantity necessary to decompose 1 μM H_2_O_2_ in 1 m. Enzyme activity was quantified as U/g tissue protein.

### 2.13. Superoxide Dismutase Assay

In the superoxide dismutase (SOD) assay, tissue homogenate or serum was mixed with 0.05 M potassium cyanide, 0.2 mM cytochrome, 1 mM xanthine, and 0.1 mM EDTA/0.05 M potassium phosphate buffer, as previously described [4]. Xanthine oxidase was then treated into the reaction mixture. After treatment, the SOD activity was assessed spectrophotometrically at 550 nm using a microplate reader, measuring the inhibition rate for cytochrome reduction via superoxide radical.

### 2.14. Glutathione Peroxidase Assay

The glutathione peroxidase (GPx) activity was assessed by mixing tissue homogenate or serum with 5 mM NADPH, 100 mM GSH, and 1 mM EDTA using the previously outlined procedure [4]. Subsequently, 1 U glutathione reductase prepared in 100 mM phosphate buffer (pH 7.0) was added. Following a 3-m incubation period, 10 mM cumene hydroperoxide was treated, and the conversion of NADPH to NADP^+^ was monitored spectrophotometrically at 340 nm. In this assay, 1 U of GPx activity corresponded to the generation of 1 μmol of NADP^+^ per milligram of protein per minute.

### 2.15. Lipid Peroxidation Assay

This assay was conducted following the previously outlined procedure [4], with some adjustments. Initially, 100 μL of tissue homogenate or serum was mixed with 30 μL of a prooxidative solution (250 μmol/L FeSO_4_), and 100 mM Tris–HCl buffer (30 μL, pH 7.4). The volume was then adjusted to 300 μL with water and incubated at 37 °C for 1 h. The color reaction was initiated by adding 300 μL of 8.1% sodium dodecyl sulfate (SDS). Subsequently, 500 μL of 0.8% TBA and 500 μL of acetic acid/HCl (pH 3.4) were added. The resulting mixture was incubated for 1 h at 100 °C. The produced TBA-species were confirmed at 532 nm, and their absorbance was compared with a standard curve prepared using malondialdehyde (MDA).

### 2.16. Nitric Oxide Assay

The measurement of nitric oxide metabolites in serum or tissue homogenate was performed using the Griess reaction as previously described [4]. Initially, the sample (100 μL) was treated with Griess reagent (consisting of 1% sulfanilamide, 2.5% polyphosphoric acid, and 0.1% naphthyl ethylenediamine dihydrochloride) (100 μL), then, incubated for 10 m at room temperature. Subsequently, the absorbance was assessed at 540 nm using a microplate reader. The concentration of nitrite was determined based on the absorbance at 540 nm, utilizing sodium nitrite as the standard.

### 2.17. Biochemical Analysis

The serum concentrations of adiponectin, leptin, and insulin were determined using ELISA assays (Sigma-Aldrich, St. Louis, MO, USA). The value of homeostasis model assessment of insulin resistance (HOMA-IR) was expressed as (fasting serum insulin × fasting serum glucose)/22.5 [4]. Furthermore, the concentrations of various biomarkers in the serum, such as alanine aminotransferase (ALT), aspartate aminotransferase (AST), albumin, total protein (TP), high-density lipoprotein cholesterol (HDL), glucose, total cholesterol (TC), and triacylglycerol (TG) were determined utilizing a Vitros 250 chemistry system (Ortho Clinical Diagnostics, Raritan, NJ, USA). The levels of non-HDL (low-density lipoprotein cholesterol (LDL) and very low-density lipoprotein (VLDL) were calculated by subtracting HDL from serum TC. Additionally, the atherogenic index (AI), coronary artery index (CAI), cardiac risk ratio (CRR), and atherogenic coefficient (AC) were evaluated using the Ikewuchi & Ikewuchi equations [26].

### 2.18. Immunoblotting

Immunoblotting analysis was conducted following established protocols. HepG2 cells and liver tissues were collected, homogenized in normal saline or RIPA lysis buffer, and then centrifuged at 10,000× *g* for 15 m at 4 °C. Protein concentrations were determined using the BCA assay. Equal amounts of protein from each tissue extract were analyzed by 12% SDS–polyacrylamide gel electrophoresis and subsequently transferred to polyvinylidene difluoride membranes. Following blocking process using 5% non-fat dry milk containing 0.1% Tween 20, 150 mM NaCl, and 10 mM Tris–HCl (pH 7.5) for 1 h at room temperature, the membranes were probed with primary antibodies against CD36, LXR-α, SREBP1c, LDLR, PCSK9, HMGCR (diluted at 1:1000), and beta-actin (diluted at 1:2500) for 1 h at RT. After washing three times in TBST buffer, the membranes were incubated with secondary antibodies horseradish peroxidase-conjugated for 1 h at RT. The reaction bands were visualized using SuperSignal West Atto (Thermo Fisher Scientific, Carlsbad, CA, USA) and detected with a MicroChemi (DNR Bio-imaging Systems, Jerusalem, Israel).

### 2.19. Statistical Analysis

The statistics of this study were conducted using the previous method [4,24], employing SPSS 21 (SPSS Inc., Chicago, IL, USA). The data obtained and examined from the present study were expressed as mean ± standard deviation (SD). To assess the statistical significance of comparisons between multiple groups, we performed a post-hoc Tukey’s test by utilizing one-way analysis of variance. Statistical significance was expressed as *p*-values less than 0.05.

## 3. Results

### 3.1. Effect of ESKO and Palmitic Acid on Cell Viability in HepG2 Cells

**Figure 1:**
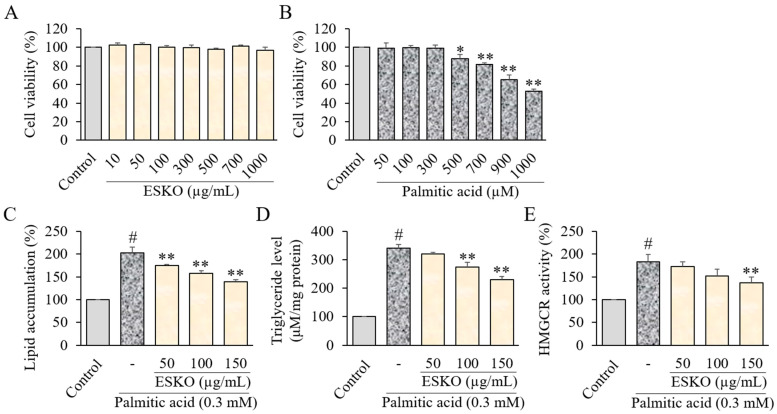
Effect of ESKO on cell viability (**A**,**B**), lipid (**C**), triglyceride (**D**) level, and HMGCR activity (**E**) in HepG2 cells. MTT assay showing the effect of ESKO and palmitic acid on HepG2 cells viability. Cells were incubated with ESKO at different concentrations (0–1000 μg/mL) or palmitic acid (0–1000 μM) for 24 h and cell viability was analyzed by MTT reduction assay. Each value is the mean ± SD of triplicate measurements. # *p* < 0.01, compared with Control group, * *p* < 0.05 and ** *p* < 0.01, compared with palmitic acid only-treated group. ESKO, *Euphausia superba* krill oil; HMGCR, 3-hydroxy-3-methylglutaryl-CoA reductase (HMGCR).

The impact of ESKO on cell viability was assessed using the MTT assay on the HepG2 cell line. Various concentrations of ESKO ranging from 10, 50, 100, 300, 500, 700, and 1000 μg/mL were applied to the cell line, along with palmitic acid concentrations ranging from 50, 100, 300, 500, 700, 900, and 1000 μM, for a duration of 24 h. While treatment with ESKO did not influence cell viability (Figure 1A), exposure to palmitic acid resulted in a decrease in cell viability at concentrations between 500–1000 μM (Figure 1B).

### 3.2. Effect of ESKO on Lipid Accumulation and HMG-CoA Reductase Activity in HepG2 Cells

Following the evaluation of intracellular fat accumulation levels, it was noted that treatment with ESKO at concentrations of 100 and 150 μg/mL led to a dose-dependent inhibition of both lipid (Figure 1C) and TG level (Figure 1D) compared to the group treated solely with palmitic acid. Additionally, a dose-dependent decrease in HMGCR activity was observed with ESKO treatment at concentrations of 100 and 150 μg/mL compared to the group treated solely with palmitic acid (Figure 1E).

### 3.3. In Silico Molecular Docking on HMG-CoA Reductase

The models of molecular docking between the enzyme and the ligands were displayed using Discovery Studio Visualizer software. Figures S1–S4 display the hydrophobicity levels and the hydrogen bonds present in the binding pockets of all ligands. Astaxanthin interact with 22 amino acids located in the cytosolic domain (residues 460–888) in which the active site of HMGCR, among which the amino acids of ALA556, THR557, ASP690, LYS691, Lys692, ALA751, HIS752, ASN755, THR758, TYR761, GLN766, ALA769, and ASN771 were bound by van der Waals interactions, unfavorable steric bumps interactions with LYS691, LYS692, ASN750, ALA753, ALA754, THR758, ALA768, ASN771, and SER775, Alkyl of hydrophobic interaction bonds with VAL757, ILE762, ALA768, ALA769, and VAL772 and a conventional hydrogen bond with THR558 in Figure S1 and Table S1. DHA interacted with 27 amino acids located in the active site of HMG-CoA reductase, among which the amino acids of LYS692, ALA751, HIS752, ASN755, ILE756, THR758, ILE760, ILE762, ALA763, GLN766, ASP767, ALA769, GLN770, ASN771, MET797, VAL846, and GLU850 form van der Waals interactions, ASN750, ALA753, ALA754, VAL757, THR758, TYR761, ALA768, ASN771, and SER775 form unfavorable steric bumps of unfavorable interaction, ILE762, ALA768, ALA769, VAL772, and CYS777 were bound by Alkyl of hydrophobic interactions, and TYR761 was bound by hydrophobic interaction (Pi-Alkyl) in Figure S2 and Table S1. EPA interact with amino acids located in the active site of HMGCR by van der Waals interactions with HIS752, ASN755, ILE756, THR758, ALA759, ILE762, ALA769, GLN770, SER775, and ILE802, a conventional hydrogen bond interaction with ASN750, unfavorable steric bumps interactions with ALA753, ALA754, VAL757, THR758, TYR761, ALA768, ALA769, ASN771, VAL772, and VAL842, Alkyl of hydrophobic interactions with LYS691, ILE760, and ALA768, and a Pi-Alkyl of hydrophobic interaction with TYR761 in Figure S3 and Table S1. Atorvastatin interacted with ALA754, ILE756, ALA769, and GLY773 by forming van der Waals, ALA753, THR758, ALA768, ASN771, and SER775 by conventional hydrogen bond, ALA768 by carbon hydrogen bond, VAL757, THR758, ALA768, ALA769, ASN771, and VAL772 by forming unfavorable steric bumps, ALA769, GLN770, and ASN771 by halogen (fluorine) of unfavorable interaction, and ILE762 by Alkyl of hydrophobic interaction in Figure S4 and Table S1. These docking results indicate that ESKO-derived astaxanthin, DHA, and EPA bind to the HMG binding site of LYS691 and ASP767 as a major active site in the cis-loop of HMGCR, and several amino acids close to GLU559, LYS691, or ASP767 in the key catalytic site.

In the binding modes (*n* = 10) analyzed by Autodock Vina for HMG-CoA reductase, ESKO-derived substances exhibited the average binding affinities were −6.9 kcal/mol (astaxanthin), −4.57 kcal/mol (DHA), −4.88 kcal/mol (EPA), and −8.17 kcal/mol (atorvastatin). The maximum binding affinities of −7.2 kcal/mol, −4.8 kcal/mol, −5.3 kcal/mol, and −8.9 kcal/mol in the same order, as shown in Table S2 and Figures S1–S4.

### 3.4. Effect of ESKO on CD36, LXRα, SREBP1c, LDLR, and PCSK9 Signaling and HMGCR Activity in HepG2 Cells

**Figure 2:**
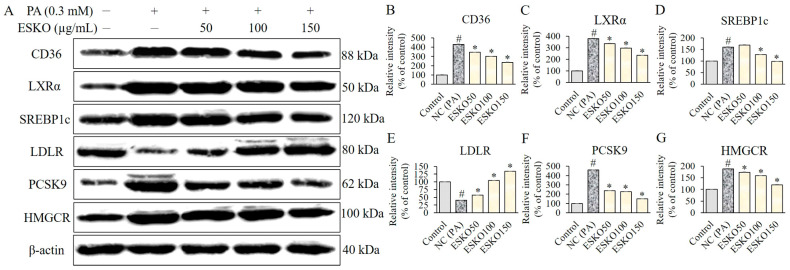
Effect of ESKO on CD36 (**A**,**B**), LXRα (**C**), SREBP1C (**D**), LDLR (**E**), and PCSK9 (**F**) signaling and HMGCR activity (**G**) in HepG2 cells. Each value is the mean ± SD of triplicate measurements. # *p* < 0.01, compared with Control group, * *p* < 0.01, compared with palmitic acid only-treated group as negative control (NC). PA, palmitic acid; ESKO, *Euphausia superba* krill oil; LXRα, liver X receptor α; SREBP1c, sterol regulatory element-binding protein 1c; LDLR, low-density lipoprotein receptor; PCSK9, proprotein convertase subtilsin/kexin type 9; HMGCR, 3-hydroxy-3-methylglutaryl-CoA reductase.

Treatment with palmitic acid significantly elevated the expression levels of PCSK9, CD36, LXRα, and SREBP1c (Figure 2A). However, upon treatment with ESKO at concentrations of 50, 100 and 150 μg/mL, the expression levels of PCSK9, CD36, LXRα, SREBP1c and the level of HMGCR decreased, whereas LDLR level was increased compared to the group treated solely with palmitic acid (Figure 2B–G). These results indicate that ESKO reduces the activated HMGCR activity, and influences the modulation of PCSK9 activation, which is implicated in LDL receptor expression, or LXRα/SREBP1c activation, which is involved in FFA receptor expression. This suggests a reduction in the initial activation of fatty acid absorption and lipid synthesis in FFA-stimulated liver cells.

### 3.5. Effect of ESKO on Body Weight Gain and Feed Efficiency in DIO Mouse Models

**Figure 3:**
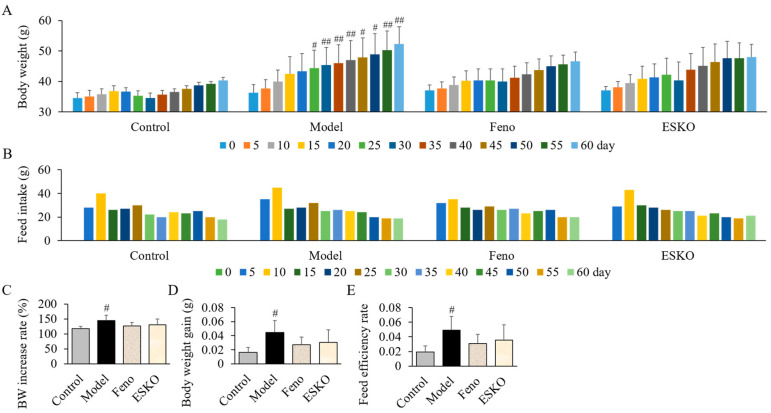
Effect of ESKO on body weight (**A**,**C**,**D**) and feed intake (**B**,**E**) in DIO mouse models. Each value is the mean ± SD (*n* = 6). # *p* < 0.05 and ## *p* < 0.01, compared with Control group. Control, non-treated normal group; Model, diet-induced obesity (DIO) model group; Feno, 200 mg/kg fenofibrate-administrated DIO group, ESKO, 400 mg/kg *Euphausia superba* krill oil-administrated DIO group.

The DIO group, which received a high-fat diet (HFD), displayed a rise in body weight from day 25 to day 60 in contrast to the Cgroup, who were fed a standard diet (Figure 3A). There were no significant differences in average body weight, feed intake, or feed efficiency rate among the ESKO and Feno groups compared to the model group (Figure 3B–E).

### 3.6. Effect of ESKO on the Weight of Tissues

Table 2 presents the assessment of ESKO and fenofibrate effects on the weight of different tissues. The kidney, spleen, and epididymal fat weights were noted to be higher in the model group compared with the Control group, whereas there were no significant alterations in liver weight. Conversely, ESKO administration resulted in reduced kidney, spleen, and epididymal fat weight gain, while the fenofibrate-administered group exhibited decreased weight gain in kidney, spleen, and epididymal fat compared to the model group.

### 3.7. Effect of ESKO on Hepatic and Epididymal Fats in DIO Mouse Models

**Figure 4:**
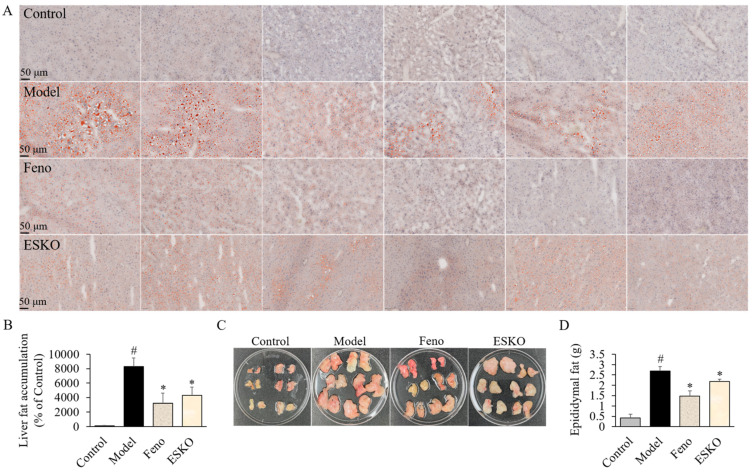
Histopathological analysis of livers and epididymal fats from DIO models. Effects of ESKO and fenofibrate on hepatic (**A**,**B**) or epididymal fats (**C**,**D**) in DIO model was analyzed with H&E, microscope, ImageJ, or fat weight. Each value is the mean ± SD (*n* = 6). # *p* < 0.01, compared with Control group, * *p* < 0.01, compared with DIO model group. Control, non-treated normal group; Model, diet-induced obesity (DIO) model group; Feno, 200 mg/kg fenofibrate-administrated DIO group, ESKO, 400 mg/kg *Euphausia superba* krill oil-administrated DIO group.

We conducted a study to investigate the effect of ESKO or fenofibrate administration on liver fat accumulation. Our analysis specifically examined hepatic fat density across different groups. As shown in Figure 4A,B, the consumption of a HFD led to a faster increase in hepatic fat accumulation compared to the Control group. However, administration of ESKO resulted in a significant reduction in fat accumulation and density compared to the model group (Figure 4B). Additionally, the weight of epididymal fat was higher in the model group compared to the Control group (Figure 4C), whereas the ESKO-administered group exhibited a decrease in epididymal fat gain compared to the model group (Figure 4D).

### 3.8. Effect of ESKO on Lipid, Total Protein, and Oxidative Stress Parameters on Liver

As depicted in Table 3, HFD feeding caused significant increases in the levels of MDA, and NOS and significant decreases in CAT, SOD, and GPx. The ESKO group demonstrated an inhibitory effect against lipid oxidation compared to that of the Model group, whereas the alterations in CAT, and GPx showed significant results (Table 3). There were no significant alterations in the levels of SOD and NOS within the ESKO group. Moreover, a HFD led to alterations in hepatic total protein and triacylglycerol. Liver TP and TG levels were elevated in the Model group compared to the Control group. In the Feno and ESKO groups, a decrease in liver TG was confirmed compared to the Model group. Notably, significant changes in MDA and CAT levels were observed in the Feno group, while there were no differences in SOD, NOS, and GPx.

### 3.9. Effect of ESKO on Oxidative STRESS Parameters in Serum

We evaluated serum oxidative stress parameters, including antioxidant enzymes, following ESKO and Feno administration in obese mice, considering the significant role of body components such as blood and liver in oxidative metabolism. As illustrated in Table 4, the Model group exhibited decreased activities of CAT, SOD, and GPx, along with increased levels of NOx and MDA compared to the Control group. There were no significant alterations in the values of MDA, CAT, SOD, NOx, and GPx within the ESKO group. The Feno group displayed an inhibitory effect against lipid oxidation and NOx, alongside a notable increase in SOD levels.

### 3.10. Effect of ESKO on Biochemical Parameters of Serum

HFD-induced obesity is closely linked to adipokines, lipids, atherogenesis, cardiac risk, and insulin resistance. Table 5 displays the serum levels of key biochemical parameters, encompassing adiponectin, leptin, proteins, lipids, insulin, glucose, and various indices. In comparison to the Control group, feeding the Model group mice an HFD for 60 days resulted in elevated levels of all indices and parameters, except for adiponectin, which exhibited a significant decrease, and significant differences were not observed in albumin, TP, HDL, and glucose values. ESKO administration decreased the values of leptin, ALT, AST, and lipid metabolic parameters such as TC, TG, and LDL + VLDL (non-HDL), while elevating the level of adiponectin. The Feno group exhibited significant reductions in TC, TG, and LDL + VLDL (non-HDL) levels and a significant decrease in AST. The atherogenic index, cardiac risk ratio, atherogenic coefficient, and coronary artery index were increased following HFD feeding compared to the Control group, as illustrated in Table 5. The increased values of atherogenic index, cardiac risk ratio, atherogenic coefficient, and coronary artery index were notably reduced in the ESKO and Feno groups compared to the Model group. In ESKO-administered obese mice, serum insulin levels and insulin resistance were significantly decreased in comparison with those of the Model group.

### 3.11. Effect of ESKO on HDF-Induced CD36/LXRα/SREBP1c, LDLR/PCSK9 Signaling and HMGCR Level in Livers of Obese Mice

**Figure 5:**
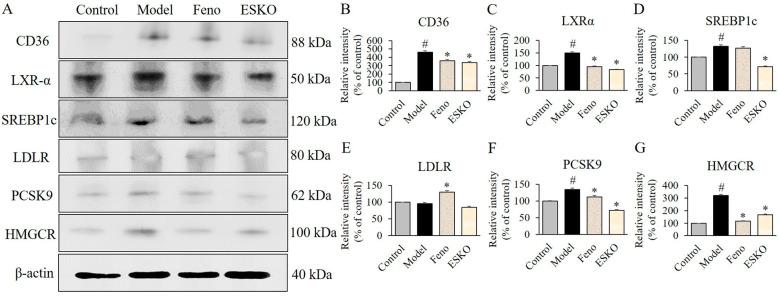
Effect of ESKO on CD36 (**A**,**B**), LXRα (**C**), SREBP1C (**D**), LDLR (**E**), and PCSK9 (**F**) signaling and HMGCR activity (**G**) in livers from DIO model. Each value is the mean ± SD (*n* = 6). # *p* < 0.01, compared with Control group, * *p* < 0.01, compared with DIO Model group. Control, non-treated normal group; Model, diet-induced obesity (DIO) Model group; Feno, 200 mg/kg fenofibrate-administrated DIO group, ESKO, 400 mg/kg *Euphausia superba* krill oil-administrated DIO group. LXRα, liver X receptor α; SREBP1c, sterol regulatory element-binding protein 1c; LDLR, low-density lipoprotein receptor; PCSK9, proprotein convertase subtilsin/kexin type 9; HMGCR, 3-hydroxy-3-methylglutaryl-CoA reductase.

We conducted further investigations to determine if ESKO administration could mitigate HDF-induced activation and elucidate its underlying mechanisms (Figure 5A). Our results revealed that inducing a HFD upregulated the protein levels of CD36, LXRα, SREBP1c, PCSK9, and HMGCR (Figure 5B–D,F,G), while we did not confirm changes in LDLR level (Figure 5E) compared to the Control group. Within the ESKO group, significant reductions were observed in the levels of CD36, LXRα, SREBP1c, PCSK9, and HMGCR compared to the Model group. However, differences in LDLR level were not significant in the ESKO administration group. Feno administration decreased CD36, LXRα, PCSK9, and HMGCR levels while increasing LDLR levels. These findings illustrate that the modulation of the underlying mechanisms following HDF-induced stimulation by mitigating the PCSK9/HMGCR or CD36/LXRα/SREBP1c pathway via ESKO intake aligns with the attenuation of FFA-caused stimulation of lipid synthesis and fatty acid oxidation in DIO models.

## 4. Discussion

In our current investigation, a high-fat diet (HFD) for 60 days induced changes consistent with prior studies, including hyperlipidemia, hepatic lipid accumulation, and heightened adiposity in the diet-induced obesity model [4,19,27,28,29]. Mice on an HFD showed a 1.45-fold increase in body weight, a 2.74-fold increase in body weight gain, a 6.53-fold increase in epididymal fat, and a 1.26-fold increase in hepatic fat mass compared to those on a normal diet. These changes may be associated with hypercholesterolemia, hypertriglyceridemia, cardiovascular risk factors, and insulin resistance. Furthermore, the HFD caused increases in TG, TC, LDL + VLDL, insulin, insulin resistance, cardiovascular risk indicators including AC, AI, CAI, and CRR, and glucose. It also resulted in reduced adiponectin levels and elevated serum leptin levels in the diet-induced obesity mouse models. However, ESKO administration proved to be more effective than fenofibrate (lipid-lowering drug), as it significantly reduced leptin levels and increased adiponectin levels. The anti-obesity efficacy of ESKO might stem from its rich composition of potent antioxidants like vitamins A and E, along with various bioactive compounds such as omega-3 polyunsaturated fatty acids (PUFAs), notably docosahexaenoic acid (DHA) and eicosapentaenoic acid (EPA), as well as astaxanthin and 6,8-di-C-glucosylluteolin [13,28]. These constituents are renowned for their antidiabetic, anti-obesity, antioxidant, anti-inflammatory properties, and for reducing the risk of cardiovascular diseases (CVDs) [13,14]. Multiple studies have shown that krill oil treatment inhibited the risk of atherosclerosis by decreasing the expression of pro-inflammatory genes including intercellular adhesion molecule 1, vascular cell adhesion protein 1, chemokine C-C motif ligand 19, and chitinase-3-like 1 in arterial endothelial cells [15], facilitates the reduction of plasma lipids, and regulates lipid balance by decreasing the expression of genes associated with the initial stages of isoprenoid/cholesterol and lipid synthesis [16], its supplementation suppressed HMGCR activity, and stimulated AMPK phosphorylation, LDL receptor and ACAT2 expression in the liver [17]. Moreover, krill oil ameliorates high-fat diet-induced cognitive impairment by reducing oxidative stress in the brain, including reactive oxygen species (ROS), lipid peroxidation, and nitric oxide (NO) [18], and phospholipids from Antarctic krill stimulate the expression of fatty acid synthetase (FAS), and peroxisome proliferator-activated receptor alpha (PPAR-α) in liver tissue. Additionally, they enhance the expression of tight junction genes such as ZO-1 and Occludin in colon tissue [19]. In addition, several studies have reported the antihyperlipidemic effects of krill oil using animal models and cell studies [27,30,31].

One notable discovery from our study is the significant decrease in PCSK9 levels and the upregulation of LDLR levels in HepG2 cells or the livers of obese model mice treated with ESKO. This suggests that ESKO administration inhibits PCSK9, thereby promoting the activation of LDLR in the liver. The interaction between elevated free fatty acids (FFA) and CD36 triggers the activation of the CD36-LXR-SREBP1c pathway. Our observation of reduced levels of CD36 and SREBP1c indicates that ESKO administration mitigated hepatic lipogenesis and lipid uptake. The SREBP family transcriptionally controlled adipogenesis. SREBP1c and SREBP2 share some functional similarities; however, SREBP1c primarily regulates fatty acid synthesis or LDLR expression-related genes, while SREBP2 mainly controls the genes expression associated with cholesterol biosynthesis [32]. This coordinated activation of adipogenic transcription promotes adipocyte maturation. Atherosclerosis is an arterial condition marked by cholesterol accumulation, which leads to chronic low-grade inflammation and the formation of cholesterol-rich foam cells [33]. Reduced LDLR expression in the early stages of foam cell formation occurs as a result of elevated cellular cholesterol, and this process is initiated via the interaction of CD36 with oxidized LDL. Lipid efflux within foam cells is supported via several macrophage transport proteins [33]. SIRT6 inhibits SREBP transcriptional activity and following cleavage for activation, leading to a reduction in cholesterol synthesis [33]. In cholesterol biosynthesis, the liver releases VLDL into the bloodstream, and these VLDL molecules are subsequently transformed into LDL, which is then absorbed by LDL receptors in peripheral cells. Within the endoplasmic reticulum, cellular cholesterol undergoes sensing, transportation, or esterification following its uptake [34]. In order to diminish intracellular cholesterol, ApoA-I aids in the transport of phospholipids and cholesterol through ATP-binding cassette (ABC) transporters, forming HDL particles [34]. When there is a cholesterol surplus, the spare cholesterol may become 22-hydroxycholesterol, which activates LXRs, thereby increasing the ABC transporter expression and promoting cholesterol efflux [33]. The interaction between PCSK9 and LDL metabolism is bidirectional. Inhibition of intracellular cholesterol biosynthesis, such as through statin use, leads to the upregulation of PCSK9, and this upregulation occurs through the recruitment of SREBP1 and SREBP2, which bind to the SRE in the proximal promoter region of the gene [35]. Dietary saturated fatty acids (SFAs) increase SREBP2, leading to elevated PCSK9 expression, while n-3 PUFA downregulate SREBP2, likely by modulating the phosphorylation of mitogen-activated protein kinases [36]. Additionally, n-6 PUFA administration has been shown to reduce PCSK9 expression by mitigating inflammatory responses, possibly through the reduction of interleukin-1 and tumor necrosis factor receptor 2 (TNF-R2) expression, which in turn upregulate SREBP2 [37]. The fasting state is associated with decreased PCSK9 levels and cholesterol biosynthesis, likely due to downregulation of SREBP2 [38]. Moreover, PUFAs have the ability to activate peroxisome proliferator-activated receptors α (PPARα), which results in the suppression of PCSK9 promoter activity and SREBP1c expression [39]. In contrast to the regulation of SREBP and PCSK9, inducible degrader of LDLR (IDOL) regulation is governed by the sterol-responsive nuclear receptors LXRα and LXRβ, which are involved in maintaining LDLR stability [40]. In particular, activation of LXRs prevents cellular cholesterol accumulation by promoting the ubiquitin-mediated degradation of LDLR via IDOL and increases the expression of genes involved in promoting cholesterol efflux [41].

Oxidative processes play a role in the onset of obesity. Adipokines produced by white adipose tissue contribute to the generation of reactive oxygen species (ROS), leading to oxidative stress through mechanisms such as the production of free radicals due to excessive consumption of lipid-rich diets, oxygen consumption, and peroxisomal or mitochondrial fatty acid oxidation [42,43]. Furthermore, oxidative stress and obesity are linked to the development of metabolic syndromes, including diabetes, insulin resistance, ischemic heart diseases, systemic arterial hypertension, asthma, gout, obstructive sleep apnea, psychological issues, rheumatological complications, liver failure, peripheral vascular disease, and oncological issues [4]. Persistent symptoms of obesity progressively diminish antioxidant capacities, resulting in decreased activity of antioxidant enzymes like CAT, SOD, and GPx, ultimately leading to complications associated with obesity [4]. Moreover, biomarkers utilized for obesity and oxidative stress assessment, NO and MDA, have been reported [4,44]. Elevated production of endothelial NO and superoxide in obese individuals may elevate peroxynitrite levels, impairing the efficacy of NO and inducing vasoconstriction in hepatic blood vessels [45]. Conversely, reduction in body fat is known to bolster antioxidant capacities and improve oxidative parameters. These effects are attributed to the intake of essential antioxidant compounds such as vitamin E, vitamin C, and flavonoids [4]. In the findings of the present study, ESKO contains astaxanthin (244 mg/kg) with potent antioxidant properties [46,47,48]; animal experiments demonstrated its ability to mitigate lipid peroxidation in the liver of obese models and delay the decline in catalase and glutathione peroxidase enzyme activities. Although an inhibitory effect was confirmed in average serum levels of obese models, no significant changes were observed. Antioxidants are substances that impede oxidation, a chemical process that generates unstable free radicals capable of damaging cells and contributing to obesity and diverse ailments. Intake of antioxidants has been associated with diminished levels of oxidative stress and inflammation, both pivotal in the onset and advancement of obesity [49,50].

Other notable findings from our experiment include in silico molecular docking results between HMGCR and ESKO-derived active substances, and the significant reduction in HMGCR levels in HepG2 cells or the livers of obese model mice treated with ESKO. HMGCR catalyzes the conversion of HMG-CoA to mevalonic acid. Consequently, the reduced HMGCR activity lowers cellular cholesterol levels, which activates SREBP-mediated signaling pathways. HMGCR Class I contains a transmembrane domain spanning residues 1 to 339, a cytosolic domain with the active site located between residues 460–888, and a linker region made up of residues 340–459 [51]. The enzyme’s core active site, responsible for reducing HMG-CoA, is found within the cis-loop and includes key catalytic residues such as GLU559, LYS691, LYS735, ASP767, and HIS866 [52]. The CoA binding site is positioned in the L-domain, where the ADP moiety of CoA binds near the surface of the enzyme in a pocket surrounded by positively charged residues [53]. The residues forming the CoA binding pocket are SER565, ASN567, ARG568, LYS722, SER865, HIS866, and TYR479. NADPH interacted with the S-domain of the neighboring subunit, where the HMG-CoA binding pocket is located. The residues SER626, ARG627, PHE628, ASP653, MET655, GLY656, MET657, ASN658, and VAL805 are part of the S-domain, while residues ASN870 and ARG871 are contributed by the adjacent monomer. When NADPH is present, a conformational change occurs in the enzyme’s C-terminus, causing the active site to close [54]. Statins contain rigid hydrophobic groups covalently linked to the HMG-like moiety. Specifically, atorvastatin, an HMGCR inhibitor, forms a hydrogen bond with the carbonyl oxygen atom of SER565. This interaction occurs within a shallow groove between helices Lα1 and Lα10, which includes residues such as HIS752 and ASN755 [55]. In this study, atorvastatin demonstrated a maximum binding affinity of −8.9 kcal/mol and interacted with 13 amino acids (ALA753, ALA754, ILE756, VAL757, THR758, ILE762, ALA768, ALA769, GLN770, ASN771, VAL772, GLY773, SER775) located within the catalytic site of HMGCR. These interactions included hydrogen bonds, van der Waals forces, steric clashes, hydrophobic alkyl interactions, and unfavorable interactions with halogens (fluorine). Astaxanthin, DHA, and EPA displayed maximum binding affinities of −7.2, −4.8, and −5.3 kcal/mol, respectively. These compounds may affect enzymatic activity by forming strong van der Waals interactions (with residues ILE756 and ALA769), unfavorable steric bonds (with residues VAL757, THR758, ALA768, ALA769, ASN771, and VAL772), and hydrophobic alkyl interactions (with residue ILE762), similar to the binding site interactions of atorvastatin.

## 5. Conclusions

ESKO has shown promising results in reducing obesity and related metabolic parameters, and as a result, it is believed that it can be used as a natural material to prevent and improve obesity and fat/cholesterol accumulation. However, further research is required to fully characterize the mechanisms underlying its efficacy and to test optimal dosages and modes of administration.

## Figures and Tables

**Table 1:** Composition of the experimental diets.

Composition	Control	Model	Feno	ESKO
	*w*/*w*			
Nitrogen-free extract	59.5	37.5	37.5	37.5
Fat	4.5	34.9	34.9	34.9
Protein	20.1	23.6	23.6	23.6
Fiber	4.6	-	-	-
Ash	5.8	-	-	-
Mineral mixture	3.5	3	3	3
Vitamin mixture	1	1	1	1
Fenofibrate	-	-	0.91	-
ESKO	-	-	-	-
	-	-	-	1.86
Protein calories	21.0	18.1	18.1	17.7
Fat calories	13.7	61.6	61.6	62.4
Carbohydrates calories	65.3	20.3	20.3	19.9
Energy (kcal/g)	4.04	4.65	4.65	4.66

The diets composition according to LabDiet 5L79 and TestDiet 58Y1. Feno, 200 mg/kg/day fenofibrate-administrated model group; ESKO, 400 mg/kg/day *Euphausia superba* krill oil-administrated model group.

**Table 2:** Effect of *Euphausia superba* krill oil and fenofibrate on several tissues, and epididymal fat weights in high-fat diet-induced obese mouse models.

Parameters	Control	Model	Feno	ESKO
Liver (g)	2.01 ± 0.12	2.52 ± 0.36	2.32 ± 0.34	2.51 ± 0.31
Kidney (g)	0.427 ± 0.010	0.472 ± 0.025 ^#^	0.422 ± 0.008 *	0.432 ± 0.012 *
Spleen (g)	0.126 ± 0.002	0.137 ± 0.003 ^#^	0.120 ± 0.003 *	0.128 ± 0.003 *
Epididymal fat (g)	0.41 ± 0.20	2.69 ± 0.22 ^#^	1.47 ± 0.26 *	2.18 ± 0.12 *

The data are presented as means ± SD, *n* = 6. One-way ANOVA followed by the post hoc Tukey test. ^#^ *p* < 0.01, compared to Control group. * *p* < 0.01, compared to Model group. Feno, 200 mg/kg/day fenofibrate-administrated model group; ESKO, 400 mg/kg/day *Euphausia superba* krill oil-administrated model group.

**Table 3:** Effects of *Euphausia superba* krill oil and fenofibrate on parameters of oxidative stress, lipid, and protein in liver from high-fat diet-induced obese mouse models.

Parameters	Control	Model	Feno	ESKO
MDA	1.33 ± 0.04	1.82 ± 0.06 ^#^	1.59 ± 0.04 **	1.50 ± 0.05 **
CAT	35.22 ± 2.45	22.51 ± 1.98 ^#^	30.43 ± 2.73 *	30.19 ± 2.96 *
SOD	0.65 ± 0.02	0.42 ± 0.03 ^#^	0.44 ± 0.03	0.48 ± 0.02
NOS	2.04 ± 0.05	2.27 ± 0.09 ^#^	2.24 ± 0.03	2.24 ± 0.04
GPx	0.23 ± 0.01	0.17 ± 0.01 ^#^	0.20 ± 0.01	0.21 ± 0.02 *
Liver TG	0.39 ± 0.03	0.73 ± 0.05 ^#^	0.53 ± 0.03 **	0.59 ± 0.05 *
Liver TP	31.5 ± 1.7	40.9 ± 0.8 ^#^	33.6 ± 2.0 **	36.3 ± 1.9

The data are presented as means ± SD, *n* = 6. One-way ANOVA followed by the post hoc Tukey test. ^#^ *p* < 0.01, compared to Control group. * *p* < 0.05 and ** *p* < 0.01, compared to DIO group. Feno, 200 mg/kg/day fenofibrate-administrated model group; ESKO, 400 mg/kg/day *Euphausia superba* krill oil-administrated model group.; MDA, malondialdehyde (nM/mL); CAT, catalase (U/mg protein); SOD, superoxide dismutase (U/mg protein); NOS, nitric oxide synthesis (nM/min/mg protein); GPx, glutathione peroxidase (U/mg protein); TG, triacylglycerol (mg/dL); TP, total protein (mg/dL).

**Table 4:** Effects of *Euphausia superba* krill oil and fenofibrate on parameters of oxidative stress in serum from high-fat diet-induced obese mouse models.

Parameters	Control	Model	Feno	ESKO
MDA	1.37 ± 0.08	2.41 ± 0.11 ^#^	1.88 ± 0.13 **	2.12 ± 0.17
CAT	142.2 ± 5.1	128.5 ± 3.3 ^#^	135.9 ± 2.5	134.7 ± 2.1
SOD	96.3 ± 3.0	81.7 ± 1.9 ^#^	89.6 ± 1.7 *	87.0 ± 2.5
NOx	9.03 ± 0.31	11.02 ± 0.28 ^#^	10.19 ± 0.33 *	10.28 ± 0.25
GPx	13.65 ± 0.94	11.05 ± 0.41 ^#^	12.37 ± 0.31	11.97 ± 0.44

The data are presented as means ± SD, *n* = 6. One-way ANOVA followed by the post hoc Tukey test. ^#^ *p* < 0.01, compared to Control group. * *p* < 0.05 and ** *p* < 0.01, compared to DIO group. Feno, 200 mg/kg/day fenofibrate-administrated model group; ESKO, 400 mg/kg/day *Euphausia superba* krill oil-administrated model group; MDA, malondialdehyde (nM/mL); CAT, catalase (U/mg protein); SOD, superoxide dismutase (U/mg protein); NOx, nitrite (nM/mL); GPx, glutathione peroxidase (mU/mg protein).

**Table 5:** Effects of *Euphausia superba* krill oil and fenofibrate on serum metabolic parameters from high-fat diet-induced obese mouse model.

Parameters	Control	Model	Feno	ESKO
Adiponectin	5.92 ± 0.21	5.15 ± 0.18 ^#^	5.28 ± 0.16	5.66 ± 0.23 *
Leptin	5.51 ± 0.41	11.08 ± 0.65 ^#^	10.34 ± 0.42	9.42 ± 0.31 *
Albumin	2.45 ± 0.22	2.69 ± 0.08	2.39 ± 0.21	2.51 ± 0.26
TP	5.31± 0.43	5.36 ± 0.33	5.24 ± 0.35	5.37 ± 0.39
ALT	28.5 ± 1.9	37.3 ± 2.2 ^#^	36.1 ± 2.4	29.7 ± 2.1 *
AST	93.6 ± 2.7	151.3 ± 5.5 ^#^	127.3 ± 4.9 **	109.2 ± 4.1 **
TC	97.2 ± 3.9	295.2 ± 40.6 ^#^	145.0 ± 26.4 **	222.8 ± 31.5 **
TG	74.9 ± 22.0	469.3 ± 98.4 ^#^	111.7 ± 19.0 **	210.0 ± 86.7 **
LDL + VLDL	4.82 ± 10.30	221.3 ± 34.7 ^#^	55.31 ± 21.81 **	141.9 ± 32.8 **
HDL	92.3 ± 13.1	73.9 ± 12.6	89.7 ± 12.3	80.9 ± 14.3
AI	0.84 ± 0.34	6.40 ± 1.15 ^#^	1.26 ± 0.26 **	2.63 ± 1.12 **
AC	0.07 ± 0.13	3.04 ± 0.56 ^#^	0.64 ± 0.38 **	1.82 ± 0.54 **
CAI	0.23 ± 0.18	4.32 ± 0.50 ^#^	0.88 ± 0.27 **	2.34 ± 0.62 **
CRR	1.07 ± 0.13	4.04 ± 0.56 ^#^	1.64 ± 0.38 **	2.82 ± 0.54 **
Insulin	6.98 ± 1.34	41.78 ± 6.87 ^#^	9.35 ± 1.60 **	13.03 ± 4.59 **
Glucose	150.0 ± 14.6	182.9 ± 21.8	161.7 ± 19.7	179.5 ± 19.3
HOMA-IR	2.59 ± 0.64	18.67 ± 2.56 ^#^	3.76 ± 0.96 **	5.91 ± 2.65 **

The data are presented as means ± SD, *n* = 6. One-way ANOVA followed by the post hoc Tukey test. ^#^ *p* < 0.01, compared to Control group. * *p* < 0.05 and ** *p* < 0.01, compared to DIO group. Feno, 200 mg/kg/day fenofibrate-administrated Model group; ESKO, 400 mg/kg/day *Euphausia superba* krill oil-administrated Model group; Adiponectin (μg/mL); Leptin (ng/mL); albumin and TP (total protein) (mg/dL); ALT, alanine aminotransferase (U/L); AST, aspartate aminotransferase (U/L); TC, total cholesterol (mg/dL); TG, triacylglycerol (mg/dL); LDL + VLDL, low-density lipoprotein cholesterol + very low-density lipoprotein cholesterol (mg/dL); HDL, high-density lipoprotein cholesterol (mg/dL); AI, atherogenic index; AC, atherogenic coefficient; CAI, coronary artery index; CRR, cardiac risk ratio; Insulin (μIU/mL); Glucose (mg/dL), HOMA-IR, homeostasis model assessment-insulin resistance.

## Data Availability

The original contributions presented in this study are included in the article. Further inquiries can be directed to the corresponding author.

## Supplementary Materials

The following supporting information can be downloaded at: https://www.mdpi.com/article/10.3390/nu16213614/s1, Figure S1. 3D molecular binding pattern (A) containing aromatic (B), hydrogen bond pocket (C) and hydrophobic pocket (D) and 3D molecular interacting pattern (E) between ligand (astaxanthin) and protein (HMG-CoA reductase). Figure S2. 3D molecular binding pattern (A) containing aromatic (B), hydrogen bond pocket (C) and hydrophobic pocket (D) and 3D molecular interacting pattern (E) between ligand (DHA) and protein (HMG-CoA reductase). Figure S3. 3D molecular binding pattern (A) containing aromatic (B), hydrogen bond pocket (C) and hydrophobic pocket (D) and 3D molecular interacting pattern (E) between ligand (EPA) and protein (HMG-CoA reductase). Figure S4. 3D molecular binding pattern (A) and interacting pattern (B) between ligand (atorvastatin) and protein (HMG-CoA reductase). Table S1. Molecular interaction of HMG-CoA reductase with ESKO-derived DHA, EPA, astaxanthin and atorvastatin. Table S2. Docking energy for the molecular binding of ESKO-derived DHA, EPA, astaxanthin and atorvastatin on HMG-CoA reductase.
