# Supplementary material for: Antarctic Krill Euphausia superba Oil Supplementation Attenuates Hypercholesterolemia, Fatty Liver, and Oxidative Stress in Diet-Induced Obese Mice
Source: Nutrients. 2024 Oct 24;16(21):3614. doi: 10.3390/nu16213614 (PMC11547309; doi:10.3390/nu16213614)
Supplement: Supplementary file 1 [file nutrients-16-03614-s001.zip › nutrients-3260704-supplementary.pdf]

nutrients MDPI

Supplementary Materials

Antarctic Krill *Euphausia superba* Oil Supplementation Attenuates Hypercholesterolemia, Fatty Liver, and Oxidative Stress in Diet-Induced Obese Mice

Jun-Hui Choi, Se-Eun Park and Seung Kim *

Department of Food Science and Nutrition, Gwangju University, Gwangju 61743, Republic of Korea
* Correspondence: seungk@gwangju.ac.kr; Tel.: +82-62-670-2718

Current file content:

Figure S1. 3D molecular binding pattern (A) containing aromatic (B), hydrogen bond pocket (C) and hydrophobic pocket (D) and 3D molecular interacting pattern (E) between ligand (astaxanthin) and protein (HMG-CoA reductase).

Figure S2. 3D molecular binding pattern (A) containing aromatic (B), hydrogen bond pocket (C) and hydrophobic pocket (D) and 3D molecular interacting pattern (E) between ligand (DHA) and protein (HMG-CoA reductase).

Figure S3. 3D molecular binding pattern (A) containing aromatic (B), hydrogen bond pocket (C) and hydrophobic pocket (D) and 3D molecular interacting pattern (E) between ligand (EPA) and protein (HMG-CoA reductase).

Figure S4. 3D molecular binding pattern (A) and interacting pattern (B) between ligand (atorvastatin) and protein (HMG-CoA reductase).

Table S1. Molecular interaction of HMG-CoA reductase with ESKO-derived DHA, EPA, astaxanthin and atorvastatin.

Table S2. Docking energy for the molecular binding of ESKO-derived DHA, EPA, astaxanthin and atorvastatin on HMG-CoA reductase.

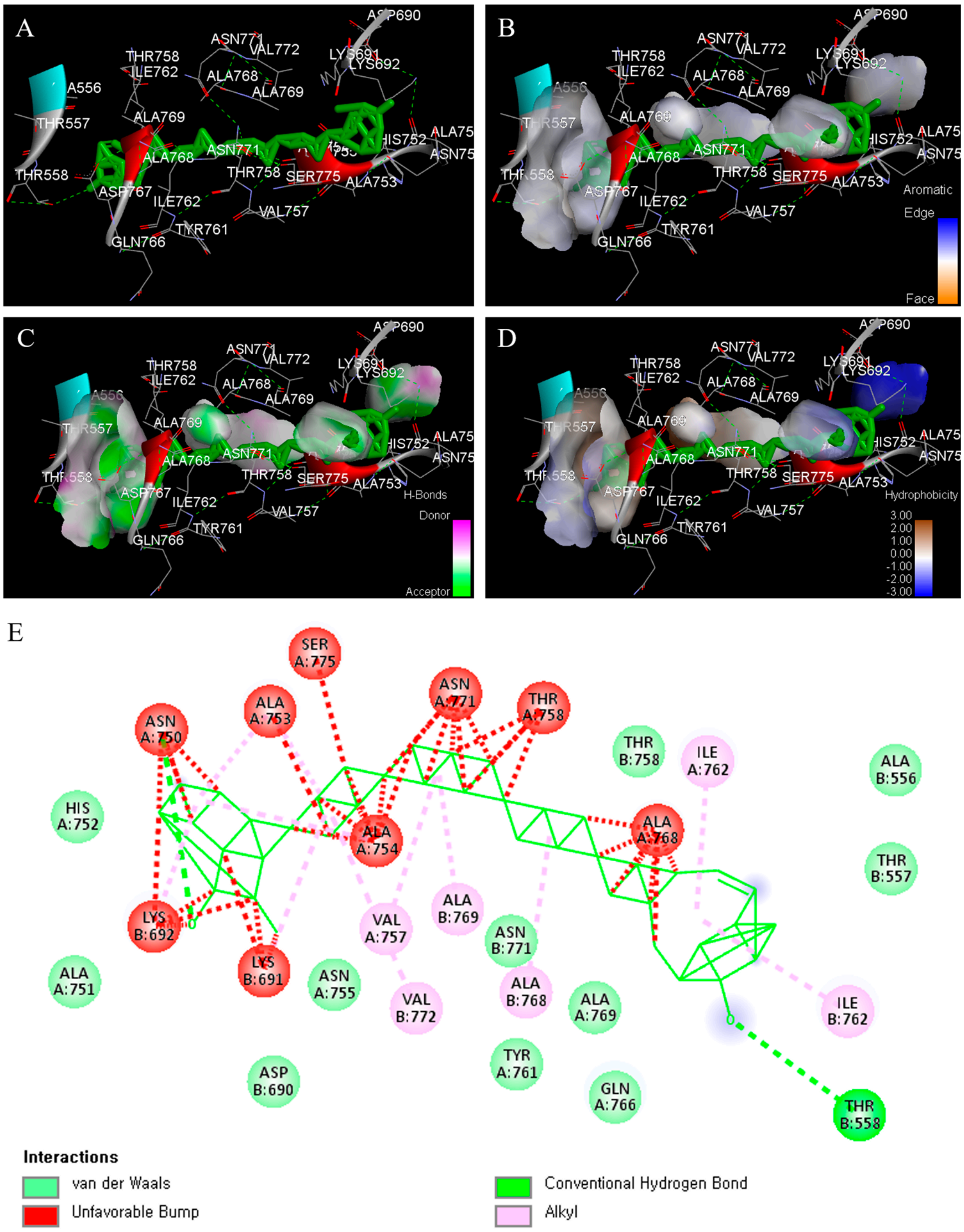

Figure S1. 3D molecular binding pattern (A) containing aromatic (B), hydrogen bond pocket (C) and hydrophobic pocket (D) and 3D molecular interacting pattern (E) between ligand (astaxanthin) and protein (HMG-CoA reductase).

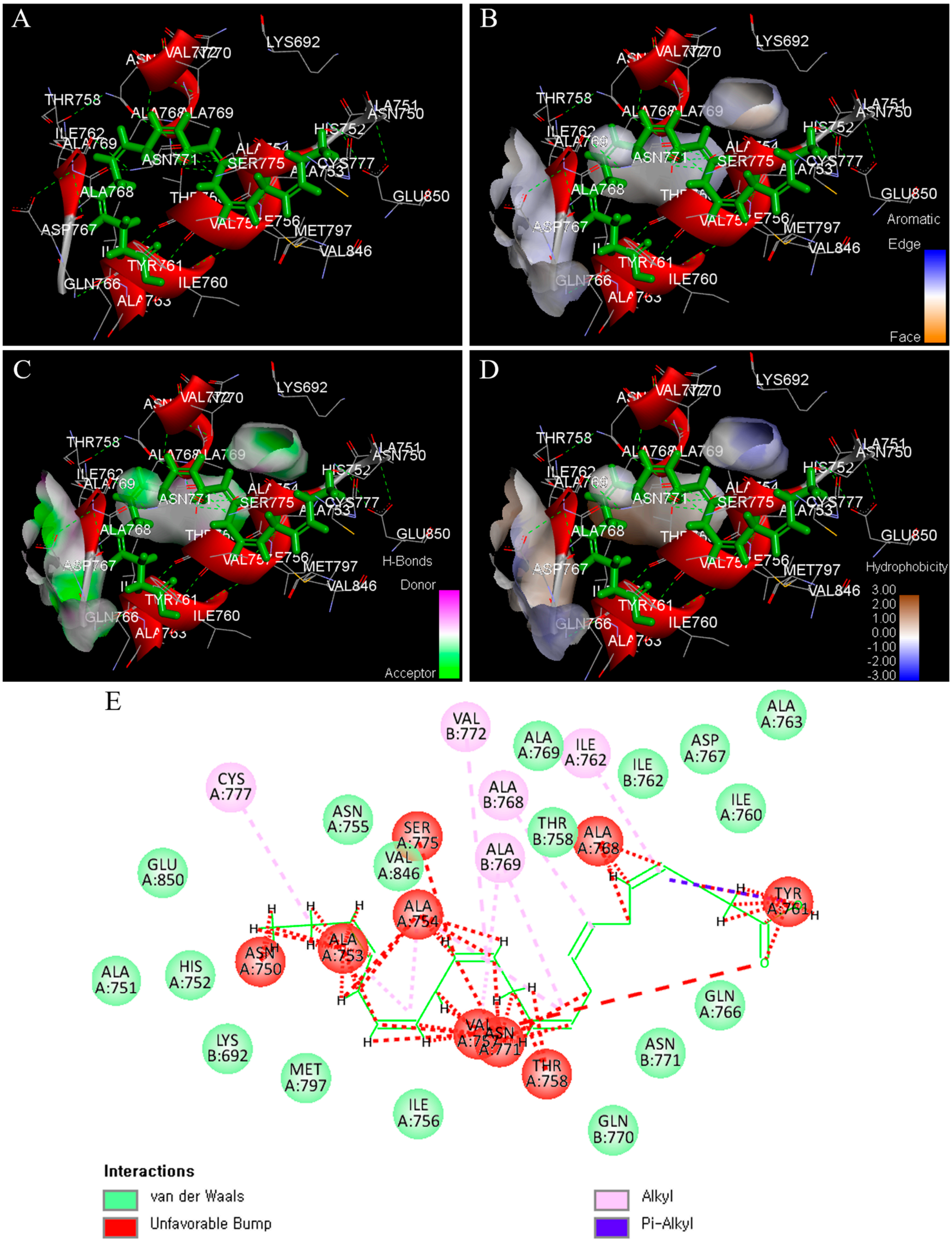

Figure S2. 3D molecular binding pattern (A) containing aromatic (B), hydrogen bond pocket (C) and hydrophobic pocket (D) and 3D molecular interacting pattern (E) between ligand (DHA) and protein (HMG-CoA reductase).

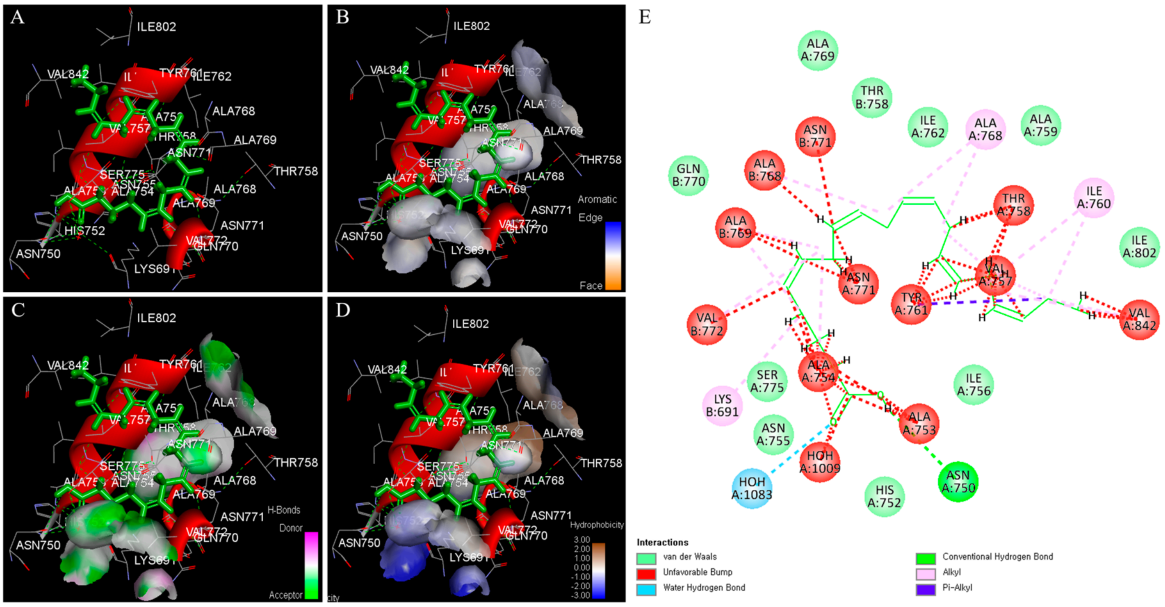

Figure S3. 3D molecular binding pattern (A) containing aromatic (B), hydrogen bond pocket (C) and hydrophobic pocket (D) and 3D molecular interacting pattern (E) between ligand (EPA) and protein (HMG-CoA reductase).

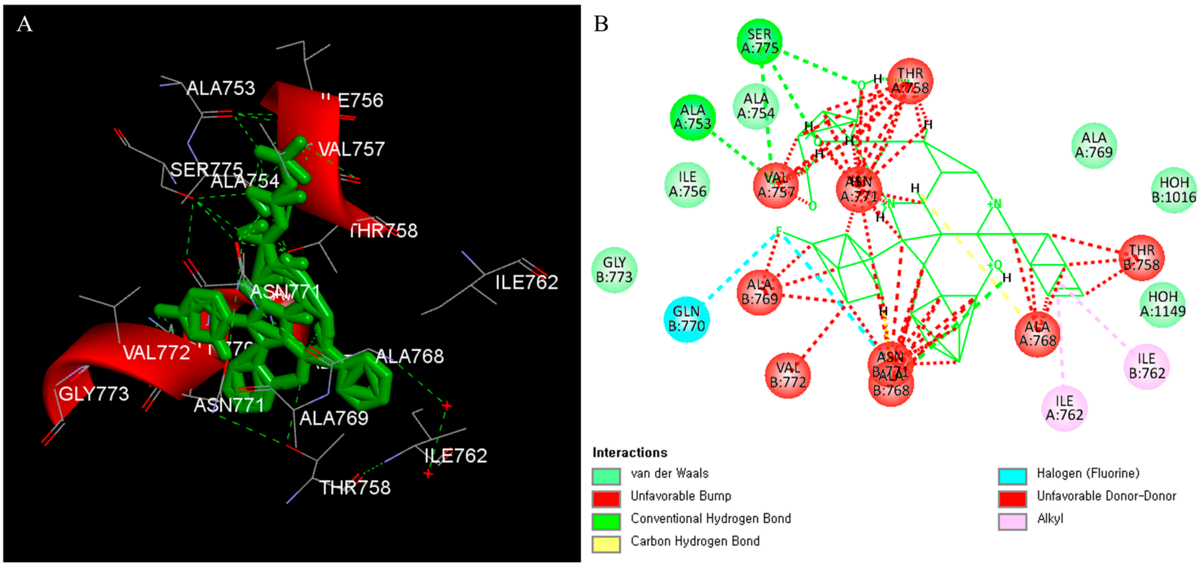

Figure S4. 3D molecular binding pattern (A) and interacting pattern (B) between ligand (atorvastatin) and protein (HMG-CoA reductase).

Table S1. Molecular interaction of HMG-CoA reductase with ESKO-derived DHA, EPA, astaxanthin and atorvastatin.

Ligand	Van der Waals	Hydrogen bond	Unfavorabale	Hydrophobic
Astaxanthin	ALA556, THR557, ASP690, ALA751, HIS752, ASN755, THR758*, TYR761, GLN766, ALA769*, ASN771*	Conventional: THR558	Steric bumps: LYS691, LYS692, ASN750, ALA753*, ALA754*, THR758*, ALA768*, ASN771*, SER775*	Alkyl: VAL757, ILE762*, ALA768*, ALA769*, VAL772*
DHA	LYS692, ALA751, HIS752, ASN755, ILE756*, THR758*, ILE760, ILE762*, ALA763, GLN766, ASP767, ALA769*, GLN770*, ASN771*, MET797, VAL846, GLU850	-	Steric bumps: ASN750, ALA753*, ALA754*, VAL757*, THR758*, TYR761, ALA768*, ASN771*, SER775*	Alkyl: ILE762*, ALA768*, ALA769*, VAL772*, CYS777 Pi-Alkyl: TYR761
EPA	HIS752, ASN755, ILE756*, THR758*, ALA759, ILE762*, ALA769*, GLN770*, SER775*, ILE802	Conventional: ASN750	Steric bumps: ALA753*, ALA754*, VAL757*, THR758*, TYR761, ALA768*, ALA769*, ASN771*, VAL772*, VAL842	Alkyl: LYS691, ILE760, ALA768* Pi-Alkyl: TYR761
Atorvastatin	ALA754, ILE756, ALA769, GLY773	Conventional: ALA753, THR758, ALA768, ASN771, SER775 Carbon hydrogen: ALA768	Steric bumps: VAL757, THR758, ALA768, ALA769, ASN771, VAL772 Halogen (Fluorine): ALA769, GLN770, ASN771	Alkyl: ILE762

* Residues matching the binding site of atorvastatin. (-) interaction, not detected.

Table S2. Docking energy for the molecular binding of ESKO-derived DHA, EPA, astaxanthin and atorvastatin on HMG-CoA reductase.

Ligand	Lowest Binding Energy (kcal/mol)	Average Docking Energy (kcal/mol)
Astaxanthin	–7.2	–6.90 (n = 10)
DHA	–4.8	–4.57 (n = 10)
EPA	–5.3	–4.88 (n = 10)
Atorvastatin	–8.9	–8.17 (n = 10)

Docking energy was analyzed by Autodock Vina. n = numbers of binding modes.
